# Acrylonitrile Derivatives against *Trypanosoma cruzi*: In Vitro Activity and Programmed Cell Death Study

**DOI:** 10.3390/ph14060552

**Published:** 2021-06-09

**Authors:** Carlos J. Bethencourt-Estrella, Samuel Delgado-Hernández, Atteneri López-Arencibia, Desirée San Nicolás-Hernández, Ines Sifaoui, David Tejedor, Fernando García-Tellado, Jacob Lorenzo-Morales, José E. Piñero

**Affiliations:** 1 Instituto Universitario de Enfermedades Tropicales y Salud Pública de Canarias, Universidad de La Laguna, Avda. Astrofísico Fco. Sánchez, S/N, 38203 La Laguna, Tenerife, Islas Canarias, Spain; cbethene@ull.edu.es (C.J.B.-E.); atlopez@ull.edu.es (A.L.-A.); dsannico@ull.edu.es (D.S.N.-H.); isifaoui@ull.edu.es (I.S.); 2 Departamento de Obstetricia y Ginecología, Pediatría, Medicina Preventiva y Salud Pública, Toxicología, Medicina Legal y Forense y Parasitología, Universidad de La Laguna, 38200 La Laguna, Tenerife, Islas Canarias, Spain; 3 Red de Investigación Colaborativa en Enfermedades Tropicales (RICET), Instituto de Salud Carlos III, 28029 Madrid, Spain; 4 Escuela de Doctorado y Estudios de Posgrado, Universidad de La Laguna, Astrofísico Francisco Sánchez, S/N, 38200 La Laguna, Tenerife, Islas Canarias, Spain; sdelgadh@ull.es; 5 Instituto de Productos Naturales y Agrobiología, Consejo Superior de Investigaciones Científicas, Avda. Fco. Sánchez 3, 38206 La Laguna, Tenerife, Islas Canarias, Spain

**Keywords:** chemotherapy, *Trypanosoma*, acrylonitrile, toxicity, Chagas

## Abstract

The neglected infection known as Chagas disease, caused by the protozoan parasite *Trypanosoma cruzi,* results in more than 7000 deaths per year, with an increasing number of cases in non-endemic areas such as Europe or the United States. Moreover, with the current available therapy, only two compounds which are active against the acute phase of the disease are readily available. In addition, these therapeutic agents display multiple undesired side effects such as high toxicity, they are expensive, the treatment is lengthy and the resistant strain has emerged. Therefore, there is a need to find new compounds against Chagas disease which should be active against the parasite but also cause low toxicity to the patients. In the present work, the activity of novel acrylonitriles against *Trypanosoma cruzi* was evaluated as well as the analysis of the physiological events induced in the treated parasites related to the cell death process. Hence, the characteristic features of an apoptosis-like process such as chromatin condensation and mitochondrial membrane potential, among others, were studied. From the 32 compounds tested against the epimastigote stage of *T. cruzi*, 11 were selected based on their selectivity index to determine if these compounds were able to induce programmed cell death (PCD) in the treated parasites. Furthermore, acrylonitriles Q5, Q7, Q19, Q27 and Q29 were shown to trigger physiological events related in the PCD. Therefore, this study highlights the therapeutic potential of acrylonitriles as novel trypanocidal agents.

## 1. Introduction

Chagas disease also known as American trypanosomiasis is a neglected infection caused by the protozoan parasite *Trypanosoma cruzi*, This disease is endemic to 21 Central and South American countries, affecting around 7 million infected people worldwide and causing more than 7000 deaths per year [1,2]. In addition, the number of reported cases in non-endemic areas has increased in recent years, reaching more than 100,000 persons in Europe and over 300,000 in the United States [3]. This increase is due to migration and novel forms of transmission of the parasite, such as infected organ transplantation, contaminated blood transfusions or congenital transmission [4].

In the endemic regions, the parasite is transmitted by its vector, which is a triatomine or kissing bug, that feeds at night and, at the same time, defecates *T. cruzi* cells in the skin of the host. At this point, the infection occurs since the trypomastigote forms penetrate the skin, breaking it or using mucosal surfaces [5].

Chagas disease, discovered by Carlos Chagas in 1909, presents two clinical phases: the acute phase is usually asymptomatic but occasionally can occur with fever, anorexia or tachycardia. This first phase is characterized by the presence of bloodstream parasites [6]; and the chronic phase, where approximately 30–40% of the patients develop some important clinical pathologies such as cardiac problems or digestive disorders (megaesophagus and megacolon), appears between 10–30 years after the acute phase. This second phase is characterized by the dissemination of the parasite by infection of different tissues of the patient. Moreover, the complication of these pathologies at this phase are the cause of death due to this infection [7,8].

The current treatment options against this disease are benznidazole and nifurtimox, which were developed 40 years ago, and which induce some important side effects such as headache, anorexia, abdominal pain, loss of weight, psychological alterations, dizziness, asthenia, nausea or vomiting, skin alterations and ataxia [9,10,11]. Usually, the first line treatment is benznidazole, with nifurtimox as the second option, because of its side effects [12]. Both treatments are active against the acute phase of the disease in children, but when the disease develops and turns to the chronic phase, or the age of the patient increases, the activity of these two drugs is reduced [13].

Recently, therapy research against *T. cruzi* has focused on the search for novel therapeutic options which also are able to induce programmed cell death (PCD) [14]. Moreover, the process of PCD found in metazoans, has been also previously described in protozoa including *T. cruzi* [15]. In addition, features such as alterations on cytoplasmic ROS accumulation levels, mitochondrial membrane potential disruption or phosphatidylserine exposure have been described in the literature, among other events conserved in parasitic protozoa [16,17].

In the last 20 years, different classes of compounds have been evaluated against *T cruzi*. Among them, it is important to mention molecules such as fexinidazole [18] or nitazoxanide [19], antifungal agents such as itraconazole [20] or posaconazole [21], antioxidants such as resveratrol [22], antidepressants such as sertraline [23], and antineoplastic agents such as imatinib [24], among others. Unfortunately, the use of these treatments is not yet recommended against the Chagas disease, because of their low efficacy and the presence of side effects. For this reason, it is important to develop new treatments presenting low toxicity and higher activity against *Trypanosoma cruzi*.

Many studies have shown that acrylonitriles possess biological activities such as antibacterial, antiparasitic or antitumoral ones. For example, this family of compounds present antibacterial activity against *E. coli*, *S. aureus* or *P. aeruginosa* [25], and also against *M. tuberculosis* [26]. Others have reported anti-acaricidal activity against *T. cinnabarinus* [27], antimalarial activity against *P. falciparum* [28], and antitumoral properties [29].

A novel group of synthetic acrylonitriles were studied in this work. Their synthesis, provided by an original process [30], delivers these structures as mixtures of (*E*,*Z*)-stereoisomers, but a simple chromatographic separation provides the pure isomers into preparative amounts. The present work tries to verify the antitrypanocidal activity of the acrylonitriles developed for this study.

## 2. Results

### 2.1. Antiparasitic Activity

The activity of the acrylonitriles were tested against the epimastigote stage of *T. cruzi*; the obtained IC_50_ are shown in Table 1.

To assess the selectivity of these compounds, their cytotoxicity was measured in a J774A.1 murine macrophage cell line, obtaining CC_50_ values, shown in Table 2.

The selectivity index (SI) was calculated as the ratio of CC_50_ value for J774A.1 and the IC_50_ value for epimastigotes of *T. cruzi*. The results are represented in Table 3.

### 2.2. Chromatin Condensation Analysis

**Figure 1:**
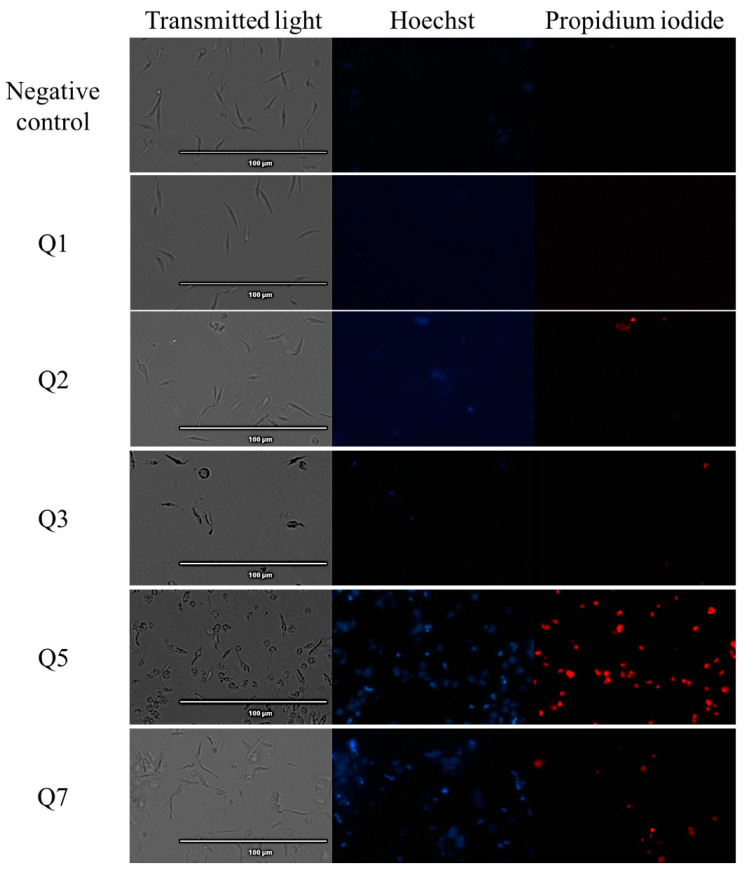

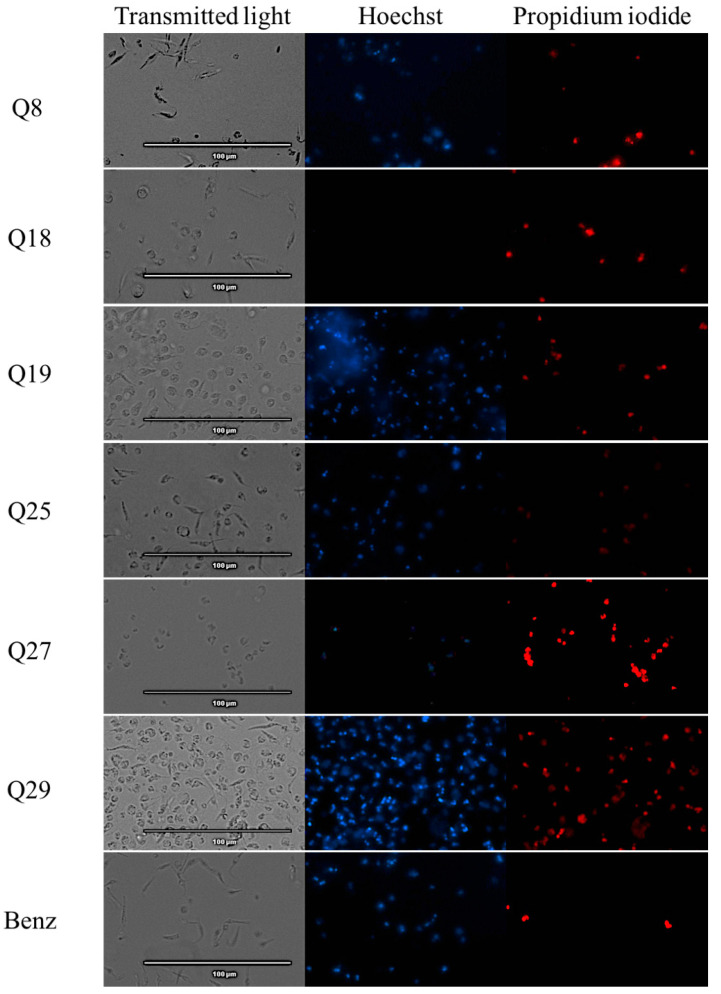
Detection of chromatin condensation using Hoechst–propidium iodide staining in treated parasites. Results after 24 h of incubation of epimastigotes against the IC_90_ of acrylonitriles. Images were captured using an EVOS FL Cell Imaging System (40×). *Scale-bar*: 100 µm. Benz: benznidazole.

The Vybran^®^ kit uses two reagents: one of them is Hoechst 33342, which shows blue fluorescence when the chromatin is condensed; the other reagent is propidium iodide, which shows red fluorescence when the parasite is dead. Figure 1 shows an intense blue fluorescence in the nucleus, indicating chromatin condensation; the images correspond to parasites treated with acrylonitriles Q5, Q7, Q19 and Q29. Some of the parasites showed red fluorescence, indicating already dead cells, which correspond with the parasites treated with acrylonitriles Q5, Q7, Q18, Q19, Q27 and Q29.

### 2.3. Mitochondrial Membrane Potential Analysis

**Figure 2:**
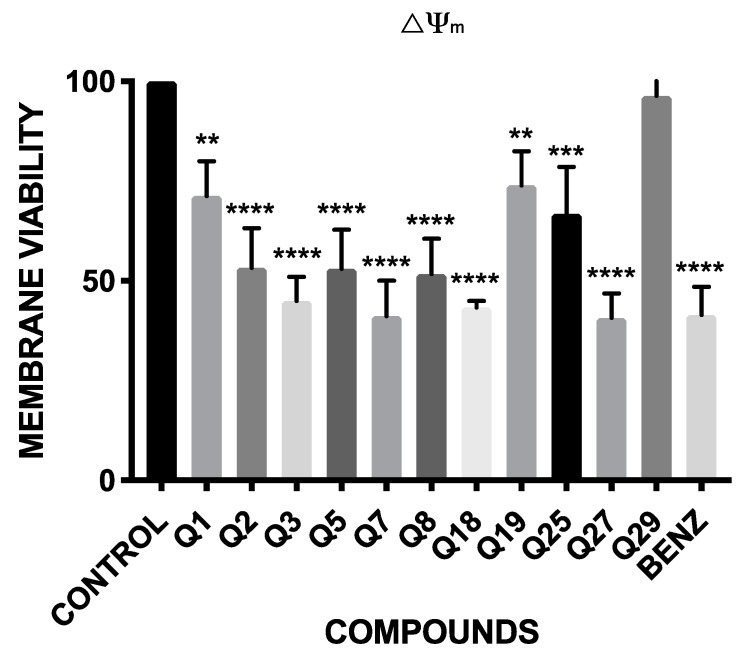
Percentage relative to control of mitochondrial membrane potential variations (ΔΨm). Benz: benznidazole. A Tukey test with the GraphPad.PRISM^®^ 7.0a soft-ware was carried out to test the statistical differences between means. (*p* < 0.05 [**]; *p* < 0.001 [***]; *p* ˂ 0.0001 [****]).

The results of the fluorescence obtained with the JC-1^®^ reagent are shown in Figure 2. These results are expressed in percentage of variations in the mitochondrial membrane potential relative to the negative control, without treatment.

The results showed that all the compounds, except Q29, presented significant variations in the mitochondrial membrane potential. These variations are highly significant in Q3, Q7, Q18 and Q27, which have a similar effect decreasing the inter-membrane potential of the mitochondria as the reference drug, benznidazole.

### 2.4. ATP Level Analysis

**Figure 3:**
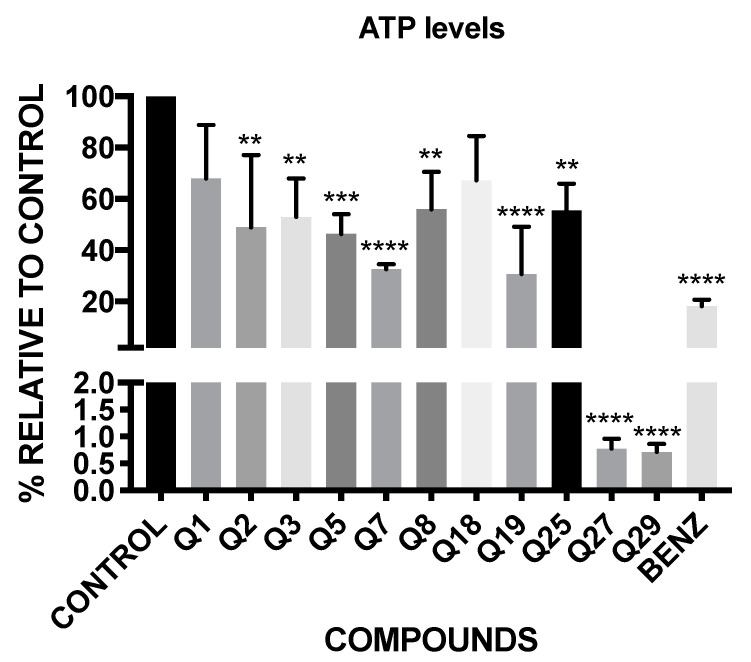
Percentage of ATP level relative to untreated control. Benz: benznidazole. A Tukey test with the GraphPad.PRISM^®^ 7.0a soft-ware test was carried out to test the statistical differences between means. (*p* < 0.05 [**]; *p* < 0.001 [***]; *p* ˂ 0.0001 [****]).

The results of the luminescence obtained with CellTiter-Glo^®^ reagent are shown in Figure 3. These results are expressed in percentage of production of ATP relative to the negative control, without treatment.

The results show that all of the tested acrylonitriles, except Q1 and Q18, have significant variations in the ATP levels of the parasites, demonstrating that Q27 and Q29 decrease the ATP levels under the 1%, continuing with Q7 and Q19, which have a percentage of ATP level of 32.68 and 30.78 respectively, close to the positive control, the benznidazole (18.26%).

### 2.5. Plasmatic Membrane Permeability Analysis

The use of the dye SYTOX^®^ Green reagent means that when the plasmatic permeability is altered, it is possible for the reagent to go inside the cells, joining the parasitic DNA and emitting an intense green fluorescence.

**Figure 4:**
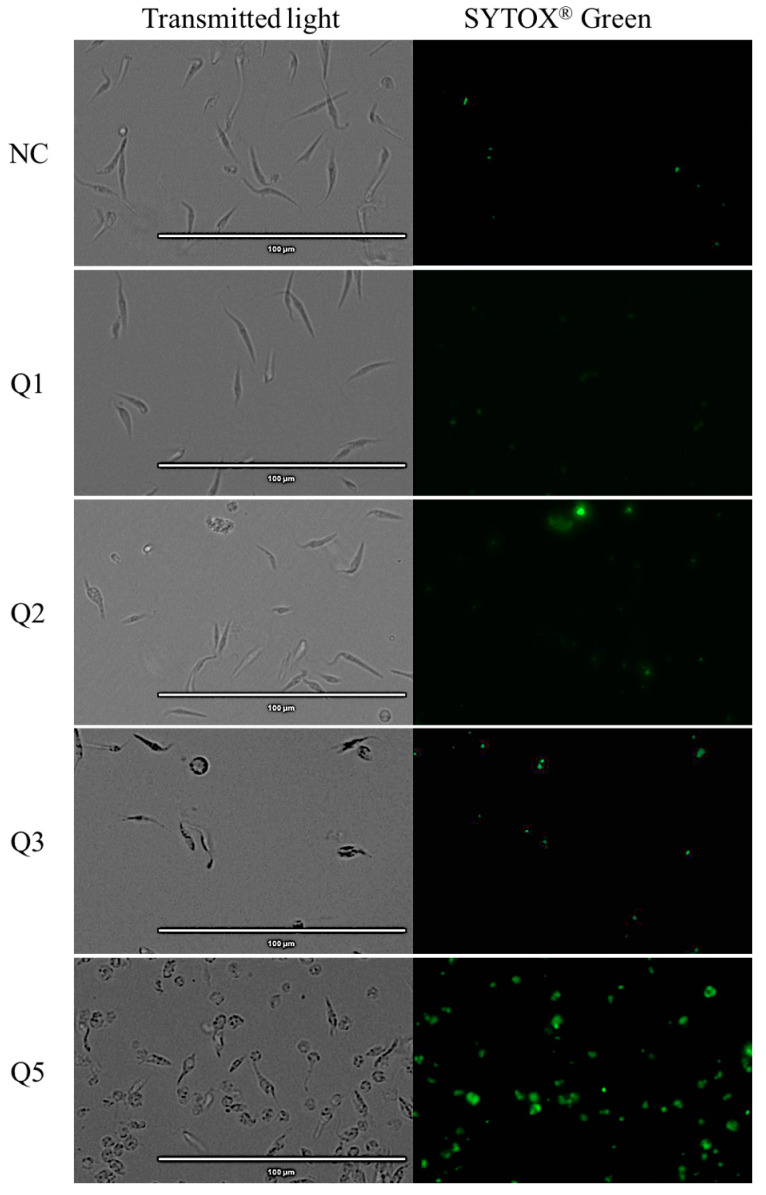

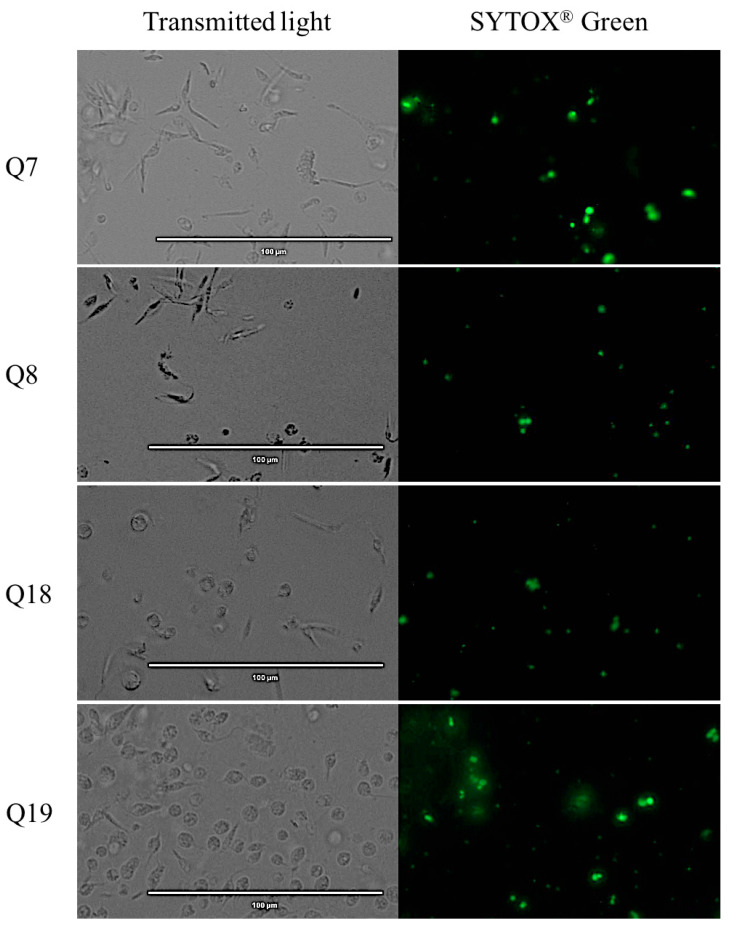

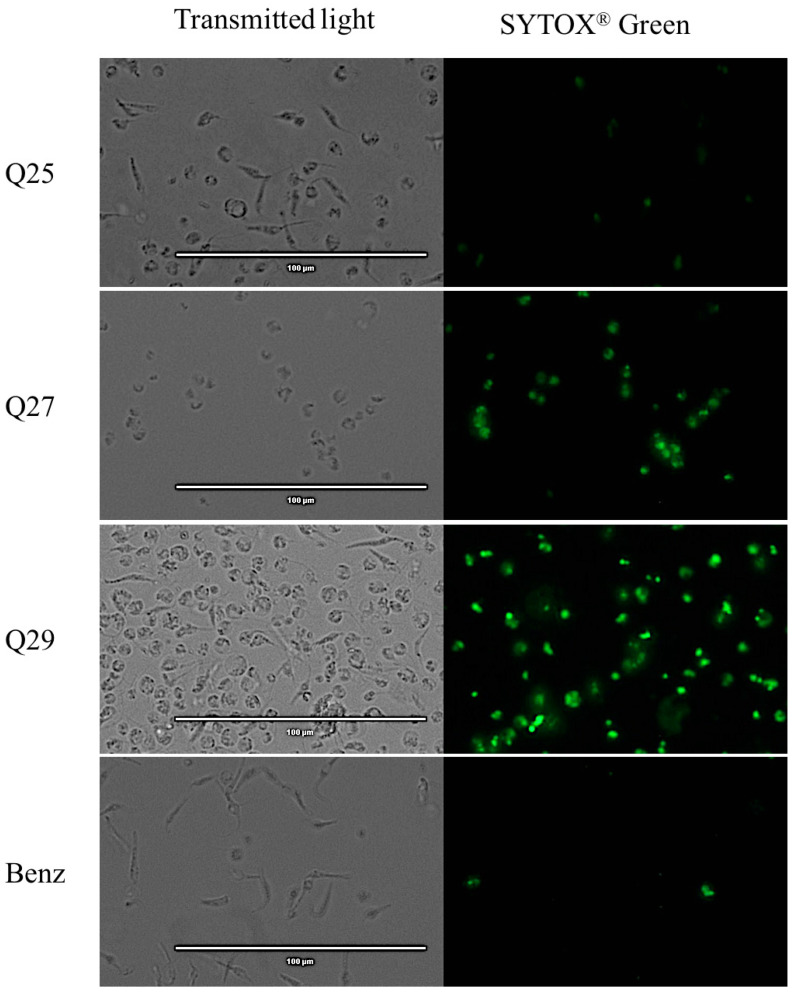
Detection of plasmatic membrane permeability by SYTOX^®^ Green staining. Epimastigotes after 24 h of incubation with the IC_90_ of the acrylonitriles. Images were captured using an EVOS FL Cell Imaging System (40×). *Scale-bar*: 100 µm. NC: negative control; Benz: benznidazole.

The images reflect that almost all acrylonitriles altered the plasma membrane permeability, demonstrated by the intense green fluorescence, and compared to the negative control which does not present this event. This intensity is more remarkable for compounds Q5, Q7, Q19, Q27 and Q29 as shown in Figure 4

### 2.6. Reactive Oxygen Species Analysis

**Figure 5:**
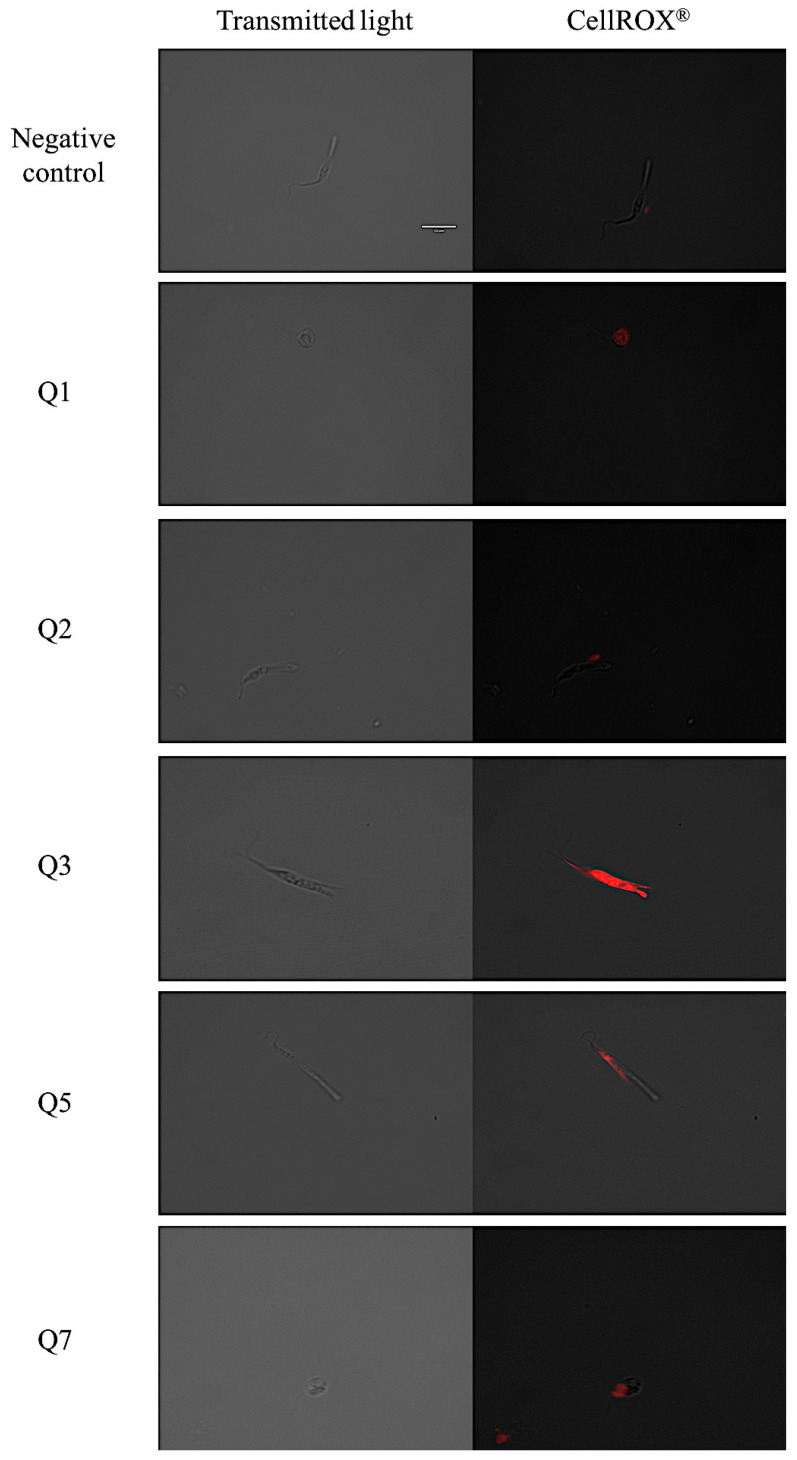

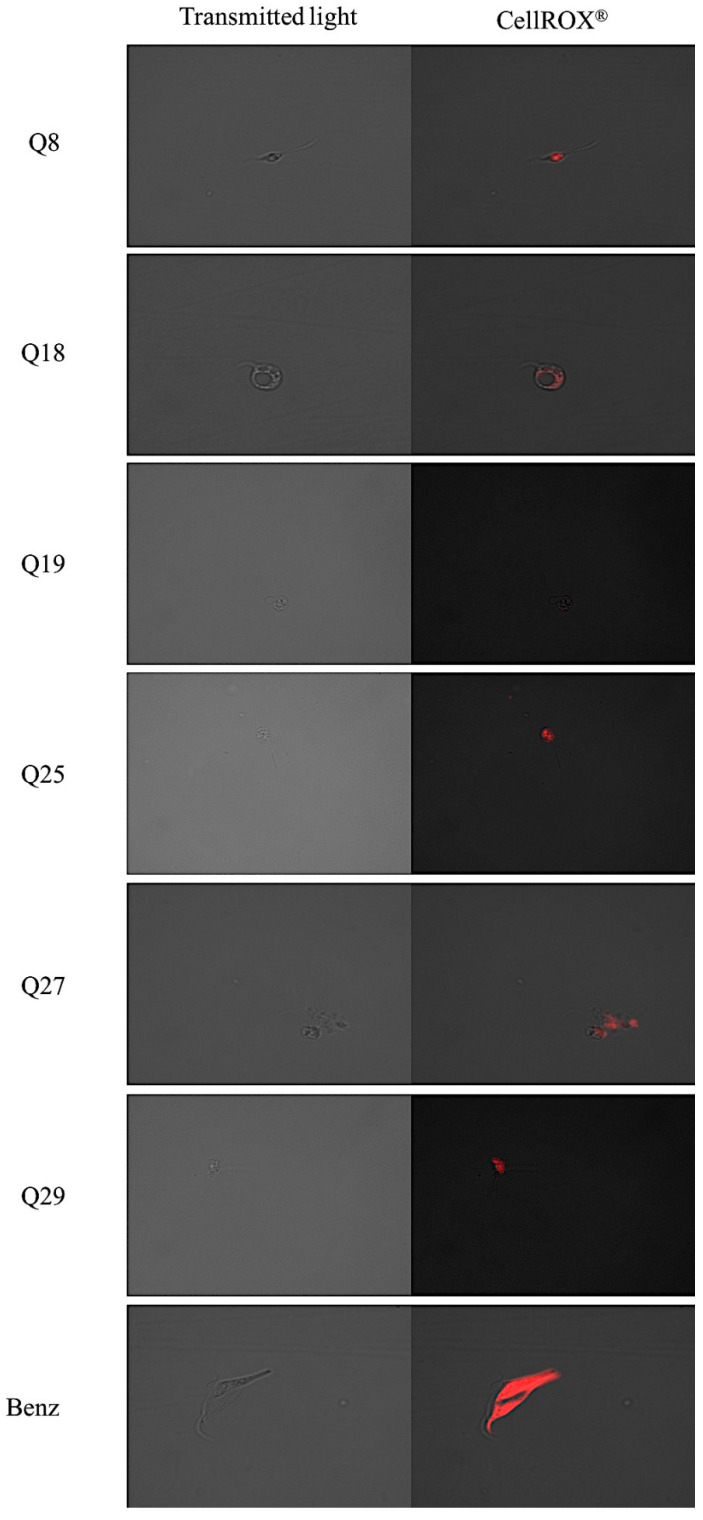
Reactive oxygen species detection by CellROX^®^ Deep Red staining. Results after 24 h of incubation with the IC_90_ of acrylonitriles. Red staining corresponds to ROS production inside the cytoplasm of the epimastigotes. Images were captured using an EVOS FL Cell Imaging System (100×). *Scale-bar*: 10 µm. Benz: benznidazole.

The CellROX^®^ reagent emits red fluorescence in the presence of reactive oxygen species. The pictures show that the most intense red fluorescence corresponds with the parasites treated with acrylonitrile Q3, while less intense fluorescence is shown in parasites treated with acrylonitriles Q5, Q7, Q8, Q18, Q27 and Q29 as shown in Figure 5.

## 3. Discussion

A limited group of compounds such as butanolides, butyrolactones, thiocarbazones, chalcones, and hydrazine derivatives, have recently been reported to present activity against *Trypanosma cruzi*, showing IC_50_ values ranging from 10.09 ± 1.5 to 590.96 ± 34.96 µM against epimastigotes [31,32,33]. Although it has been difficult to find molecules with IC_50_ values lower than 10 µM in this type of study, in the present work we obtained five compounds that achieved this goal.

From the 32 compounds evaluated in this work, 25 of them were active against the epimastigote stage of *Trypanosoma cruzi*. As shown in Table 1, the derivatives Q8, Q11, Q16, Q21 and Q32 were the most active compounds when compared to the reference drug, benznidazole, presenting IC_50_ values ranging from 3.73 ± 0.41 to 7.65 ± 1.51 µM.

The results of the present work showed that some of these compounds exhibited certain toxic effects [34,35]. However, their strong trypanocidal activities, represented by low IC_50_ values, translate into acceptable selectivity indexes, and, hence, low toxicity at the concentrations needed to eliminate the parasite. For this reason, it would be necessary to mitigate against their side effects with methodologies focusing on reducing the cytotoxicity of these compounds. These methods could include the combination of these acrylonitrile derivatives with protective drugs that present the capacity of protection against the cytotoxic damage of the acrylonitriles [36,37] or even the use of nanoparticles to reduce the cytotoxic effects of acrylonitriles [38].

Regarding the present study, acrylonitriles Q2, Q25, Q27, Q28 and Q29 were the less toxic ones, as shown in Table 2. The compounds with the optimal ratio values of cytotoxicity/activity were Q1, Q2, Q3, Q5, Q7, Q8, Q18, Q19, Q25, Q27 and Q29. Hence, the compounds with better SI values were chosen to further evaluate the induction of cell death in treated parasites.

Different assays were performed to elucidate which pathways could be triggered by these acrylonitriles. The most probable cellular process induced by our compounds is the apoptosis or programmed cell death (PCD), in which the inflammatory response is weakly activated, in contrast to a necrotic event, where the inflammatory response is strong [39]. Acrylonitriles Q5, Q7, Q19, Q27 and Q29 presented robust evidence of inducing all the physiological features of an apoptosis-like process that probably starts with a ROS accumulation, which activates the loss of mitochondrial membrane potential, and, hence, the collapse of the ATP levels, also corroborated by the chromatin condensation. On the contrary, acrylonitriles Q1, Q2, Q3 and Q25 showed good trypanocidal activity, but they did not show evidence of causing a programmed cell death mechanism [40,41,42].

Comparing the molecular structure of the acrylonitriles and their activity, we could observe different behaviors. On one hand, the acrylonitriles that have a good selectivity index are mostly *E* isomers, and only two of them are *Z* isomers. The isomers *E* and *Z* of methyl-3-cyanoacrylate (Q1 and Q2, respectively) presented a similar selectivity index, and both did not show enough evidence of induction of programmed cell death mechanisms in the parasite. However, a different phenomenon occurred when isomers *E* and *Z* of phenyl-3-cyanoacrylate (Q7 and Q8, respectively) were checked, since both types of molecules were shown to induce an apoptosis-like cell death in the treated parasites. However, the selectivity index of the *Z* isomer is more than four times higher than the one for the *E* isomer.

Interestingly, the acrylonitriles with an amide group (Q23–Q32), presented the highest values of IC_50_, representing the less active compounds of the study. In addition, the acrylonitriles with a ketone group (Q10–Q17) seemed to show the highest cytotoxic profile compared to other groups. In addition, the most selective compounds presented ester or phosphorous substituents on the acrylonitrile derivative.

## 4. Materials and Methods

### 4.1. Compounds

The compounds tested in this work (Table 4) were synthesized by our group, as previously described [30]. They were all dissolved in dimethyl sulfoxide (DMSO) (Merck, Darmstadt, Germany) and stored at −20 °C in the dark.

It is important to mention that the compounds are divided into different groups depending on the type of substituents on the acrylonitriles: esters (Q1–Q9), ketones (Q10–Q17), sulphones (Q21–Q22), amides (Q23–Q32) and phosphosubstituted (Q18–Q20).

### 4.2. Parasite Cultures

Epimastigotes of *Trypanosoma cruzi* (Y strain) were used in this study and cultured in liver infusion tryptose (LIT) medium supplemented with 10% of foetal bovine serum (FBS) at 26 °C. For the cytotoxicity assays, a murine macrophage (J774A.1) cell line was used, which was cultured in Roswell Park Memorial Institute medium (RPMI 1640, Gibco), supplemented with 10% of foetal bovine serum (FBS) at 37 °C and 5% CO_2_ atmosphere.

### 4.3. Antiparasitic Activity

In a sterile 96-well plate, a serial dilution of acrylonitriles was carried out in LIT medium supplemented with 10% FBS with a final volume of 100 µL. Parasites were added to wells to reach a concentration of 10^5^ parasites per well. Finally, a 10% of alamarBlue Cell Viability Reagent^®^ (ThermoFisher Scientific, Waltham, MA, USA) was added into each well, and the plate was incubated for 72 h at 26°C. After the incubation, the EnSpire Multimode Plate Reader^®^ (PerkinElmer, Thermo Fischer Scientific, Madrid, Spain) was used to determinate the fluorescence of each well (544 nm excitation, 590 nm emission). The concentration that inhibits 50% of the parasite population (IC_50_) was calculated by nonlinear regression analysis with 95% confidence limits [43].

### 4.4. Cytotoxicity Activity

The cytotoxicity was evaluated against murine macrophages (J774A.1) using the same method mentioned in the antiparasitic assay, based on the alamarBlue Cell Viability Reagent^®^. In a sterile 96-well plate, 10^4^ cells per well were added, then, after the complete adhesion, serial dilutions of the 32 compounds, using a deep well plate, and a 10% of alamarBlue^®^ were joined. After 24 h of incubation at 37 °C and 5% CO_2_ environment, the fluorescence was determined with the EnSpire Multimode Plate Reader^®^ to calculate the CC_50_, the concentration that inhibits 50% of the cell population [44].

### 4.5. Chromatin Condensation Analysis

Vybrant^®^ Apoptosis Assay Kit n°5, Hoechst 33342/Propidium Iodide (ThermoFisher Scientific, MA, USA) was used to determinate the chromatin condensation of the treated parasites. Epimastigotes (10^6^ cells per mL) with the IC_90_ of the compounds were incubated for 24 h at 26 °C, centrifuged (3000 rpm, 10 min, 4 °C) and resuspended in 50 µL of buffer. Thereafter, Hoechst (5 µg/mL) and propidium iodide (PI) (1 µg/mL) were added and incubated for 20 min at 26 °C. The EVOS^®^ FL Cell Imaging System (ThermoFisher Scientific, MA, USA) was used to capture the fluorescence images, using the DAPI (Hoechst) and RFP (PI) light cubes [45].

### 4.6. Mitochondrial Membrane Potential Analysis

The detection of variations in the mitochondrial membrane potential was evaluated with the JC-1 Mitochondrial Membrane Potential Assay Kit^®^ (Cayman Chemical, Ann Arbor, MI, USA). In a 24-well plate, 500 µL of epimastigotes at 10^6^ parasites per ml were added with the inhibitory concentration 90 (IC_90_) of the compounds. After 24 h of incubation at 26 °C, the epimastigotes were centrifuged (3000 rpm, 10 min, 4 °C), resuspended in 50µL of JC-1 buffer and added to a black 96-well plate with 5 µL of JC-1. After 30 min of incubation at 26 °C, the green and red fluorescence were measured using an EnSpire Multimode Plate Reader^®^ (PerkinElmer). Results were expressed in percentage relative to negative control (without treatment) of the ratio 595/535 nm (J-aggregates/J-monomers) [46].

### 4.7. ATP Level Analysis

Variations in the level of ATP were measured with the CellTiter-Glo^®^ Luminescent Cell Viability Assay (Promega, WI, USA). After 24 h of incubation with the IC_90_ of the compounds, the epimastigotes were centrifuged (3000 rpm, 10 min, 4 °C). Then, in a white 96-well plate, the epimastigotes were resuspended in 25µL of culture media and mixed with 25 µL of the kit. After 10 min of incubation at room temperature, the luminescence was measured using an EnSpire Multimode Plate Reader^®^ (PerkinElmer). The results were expressed in percentage of production of ATP level relative to the negative control (without any treatment) [47].

### 4.8. Plasmatic Membrane Permeability Analysis

To determine the plasmatic membrane permeability, SYTOX^®^ Green nucleic acid stain fluorescent dye (ThermoFisher Scientific, MA, USA) was used. A suspension of 10^6^ cells per mL of the parasites with the IC_90_ of the molecules were incubated (24 h at 26 °C) and centrifuged (3000 rpm, 10 min, 4 °C). The pellet was resuspended in a 50 µL staining buffer and the Sytox^®^ Green at 1 µM (final concentration). After 15 min of incubation at room temperature, parasites were analyzed. Fluorescence pictures were taken with the EVOS^®^ FL Cell Imaging System (ThermoFisher Scientific, MA, USA) [48].

### 4.9. Reactive Oxygen Species Analysis

The oxidative stress was measured with the CellROX^®^ Deep Red Reagent (ThermoFisher Scientific, MA, USA). After 24 h of incubation with the IC_90_ of the molecules, the parasites were centrifuged (3000 rpm, 10 min, 4 °C) and incubated with 5 μM of CellROX reagent for 30 min at 26 °C. Finally, the fluorescence was analyzed using the EVOS^®^ FL Cell Imaging System (ThermoFisher Scientific, MA, USA) with the Cy5 light cube [49].

### 4.10. Statistic Methods

The results of IC_50_ and CC_50_ are presented as the mean ± the standard deviation (SD). All the experiments were performed in triplicate on different days. The analysis were carried out using a Tukey test with the GraphPad.PRISM^®^ 7.0a software. Values of *p* < 0.05 were considered significant.

## 5. Conclusions

The studied acrylonitrile derivatives presented trypanocidal activity against *Trypanosoma cruzi* with moderate selectivity indexes, due to their moderate cytotoxic effect against murine macrophages. Several ultrastructural and morphological changes such as mitochondrial membrane potential changes, decrease in ATP levels, reactive oxygen species accumulation and chromatin condensation were observed in *Trypanosoma cruzi* epimastigotes 24 h after treatment with the mentioned acrylonitriles. The present results highlight the potential use of acrylonitrile derivatives as programmed cell death inducers on *T. cruzi*, sharing several phenotypic characteristics with other cases of programmed cell death in metazoans. However, further studies are needed to reveal the target and to decrease the cytotoxic effect of these acrylonitriles in *T. cruzi* parasites, to establish them as novel and safe trypanocidal agents.

## Figures and Tables

**Table 1:** Activity of the tested acrylonitriles against the epimastigote stage of *T. cruzi* in µM (IC: inhibitory concentration). NA: not active.

Compound	IC_50_	Compound	IC_50_	Compound	IC_50_
Q1	17.41 ± 4.75	Q12	20.94 ± 4.61	Q23	NA
Q2	25.87 ± 2.78	Q13	25.04 ± 0.62	Q24	NA
Q3	46.36 ± 5.73	Q14	26.46 ± 2.83	Q25	153.55 ± 19.79
Q4	47.3 ± 10.62	Q15	32.9 ± 0.83	Q26	NA
Q5	34.32 ± 1.67	Q16	7.65 ± 1.51	Q27	290.91 ± 46.99
Q6	51.85 ± 7.69	Q17	14.08 ± 2.53	Q28	NA
Q7	15.52 ± 1.27	Q18	14.83 ± 3.06	Q29	137.59 ± 14.01
Q8	4.36 ± 0.26	Q19	14.64 ± 3.44	Q30	NA
Q9	NA	Q20	49.13 ± 1.12	Q31	20.38 ± 0.13
Q10	11.01 ± 1.38	Q21	3.86 ± 0.67	Q32	3.73 ± 0.41
Q11	5.53 ± 1.36	Q22	NA	BENZ	6.92 ± 0.77

**Table 2:** Cytotoxicity of the tested acrylonitriles against murine macrophages (data are shown in µM). CC: cytotoxic concentration. ND: not determined.

Compound	CC_50_	Compound	CC_50_	Compound	CC_50_
Q1	87.4 ± 7.02	Q12	7.64 ± 1.21	Q23	ND
Q2	112.06 ± 15.75	Q13	8.52 ± 0.04	Q24	ND
Q3	86.65 ± 22.78	Q14	8.39 ± 0.25	Q25	374.5 ± 47.87
Q4	43.25 ± 9.83	Q15	6.66 ± 1.77	Q26	ND
Q5	53.85 ± 12.76	Q16	4.35 ± 0.25	Q27	480.44 ± 29.05
Q6	28.76 ± 2.53	Q17	5.24 ± 0.7	Q28	210.28 ± 8.99
Q7	46.45 ± 3.81	Q18	34.37 ± 1	Q29	327.87 ± 2.85
Q8	52.86 ± 4.51	Q19	22.16 ± 3.24	Q30	ND
Q9	ND	Q20	50.55 ± 10.82	Q31	10.37 ± 1.06
Q10	10.12 ± 1.46	Q21	3.19 ± 0.68	Q32	2.89 ± 0.74
Q11	5.22 ± 1.02	Q22	3.43 ± 0.68	BENZ	>1500

**Table 3:** Selectivity index (SI) of the acrylonitriles. ND: not determined.

Compound	SI	Compound	SI	Compound	SI
Q1	5	Q12	0.36	Q23	ND
Q2	4.32	Q13	0.34	Q24	ND
Q3	1.87	Q14	0.28	Q25	2.44
Q4	0.91	Q15	0.20	Q26	ND
Q5	1.57	Q16	0.57	Q27	1.65
Q6	0.55	Q17	0.37	Q28	ND
Q7	2.99	Q18	2.31	Q29	2.38
Q8	12.2	Q19	1.51	Q30	ND
Q9	ND	Q20	1.03	Q31	0.51
Q10	0.92	Q21	0.83	Q32	0.78
Q11	0.94	Q22	ND	BENZ	>222

**Table 4:** Acrylonitriles included in this study and their molecular structure.

ID	Compound	Structure	ID	Compound	Structure
Q1	(*E*)-Methyl 3-cyanoacrylate	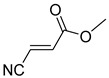	Q2	*(Z*)-Methyl 3-cyanoacrylate	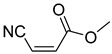
Q3	(*E*)-Ethyl 3-cyanoacrylate	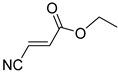	Q4	(*Z*)-Ethyl 3-cyanoacrylate	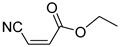
Q5	(*E*)-Octyl 3-cyanoacrylate	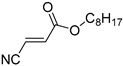	Q6	*(Z*)-Octyl 3-cyanoacrylate	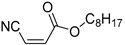
Q7	(*E*)-phenyl 3-cyanoacrylate	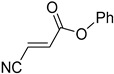	Q8	(*Z*)-phenyl 3-cyanoacrylate	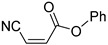
Q9	(*E*)-tert-Butyl 3-cyanoacrylate	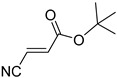	Q10	(*E*)-4-Oxonon-2-enenitrile	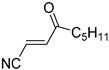
Q11	(*E*)-4-Oxo-4-phenylbut-2-enenitrile	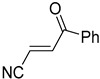	Q12	(*Z*)-4-Oxo-4-phenylbut-2-enenitrile	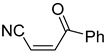
Q13	(*E*)-4-Oxo-4-(thiophen-2-yl)but-2-enenitrile	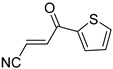	Q14	(*Z*)-4-Oxo-4-(thiophen-2-yl)but-2-enenitrile	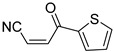
Q15	(*E*)-4-(Furan-2-yl)-4-oxobut-2-enenitrile	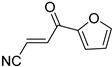	Q16	(*E*)-4-Cyclohexyl-4-oxobut-2-enenitrile	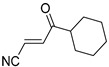
Q17	(*E*)-4-Oxo-6-phenylhex-2-enenitrile	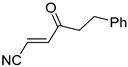	Q18	(*E*)-Diethyl 2-cyanovinylphosphonate	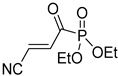
Q19	(*E*)-3-(diphenylphosphoryl)acrylonitrile	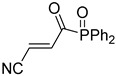	Q20	(*Z*)-3-(diphenylphosphoryl)acrylonitrile	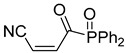
Q21	(*E*)-3-Tosylacrylonitrile	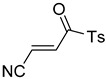	Q22	(*Z*)-3-Tosylacrylonitrile	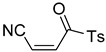
Q23	(*E*)-3-Cyano-*N,N*-dimethylacrylamide	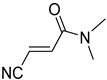	Q24	(*Z*)-3-Cyano-*N*,*N*-dimethylacrylamide	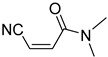
Q25	(*E*)-4-Oxo-4-(pyrrolidin-1-yl)but-2-enenitrile	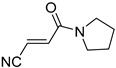	Q26	(*Z*)-4-Oxo-4-(pyrrolidin-1-yl)but-2-enenitrile	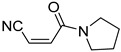
Q27	(*E*)-3-Cyano-N-methoxy-N-methylacrylamide	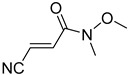	Q28	(*Z*)-3-Cyano-*N*-methoxy-*N*-methylacrylamide	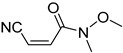
Q29	(*E*)-3-Cyano-N-methyl-N-phenylacrylamide	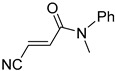	Q30	(*Z*)-3-Cyano-*N*-methyl-*N*-phenylacrylamid	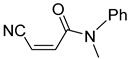
Q31	(*E*)-(*R,E*)-4-Oxo-4-(2-oxo-4-phenyloxazolidin-3-yl)but-2-enenitrile	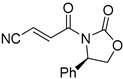	Q32	(*Z*)-(*R,Z*)-4-Oxo-4-(2-oxo-4-phenyloxazolidin-3-yl)but-2-enenitrile	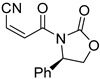

## Data Availability

The data presented in this study are available on request from the corresponding author.
